# Pre-emptive pharmacogenetic testing in the acute hospital setting: a cross-sectional study

**DOI:** 10.1093/qjmed/hcae200

**Published:** 2024-10-17

**Authors:** John H McDermott, Kerry Burke, Neil Fullerton, James O’Sullivan, Aleina Alex, Amy Ingham, Videha Sharma, Nicola Godfrey, Aghogho Odudu, Tania Syed, Andrew Stevens, Rhys Beynon, Nicholas Greaves, Daniel Akam, Selman Mirza, Paul Wilson, Stuart Wright, Katherine Payne, William G Newman

**Affiliations:** Manchester Centre for Genomic Medicine, St Mary's Hospital, Manchester University Hospitals NHS Foundation Trust, Oxford Road, Manchester, UK; Division of Evolution, Infection and Genomics, School of Biological Sciences, The University of Manchester, Manchester, UK; Division of Evolution, Infection and Genomics, School of Biological Sciences, The University of Manchester, Manchester, UK; Manchester Vascular Centre, Manchester Royal Infirmary, Manchester University NHS Foundation Trust, Manchester, UK; Manchester Centre for Genomic Medicine, St Mary's Hospital, Manchester University Hospitals NHS Foundation Trust, Oxford Road, Manchester, UK; Manchester Centre for Genomic Medicine, St Mary's Hospital, Manchester University Hospitals NHS Foundation Trust, Oxford Road, Manchester, UK; Division of Evolution, Infection and Genomics, School of Biological Sciences, The University of Manchester, Manchester, UK; Manchester Centre for Genomic Medicine, St Mary's Hospital, Manchester University Hospitals NHS Foundation Trust, Oxford Road, Manchester, UK; Manchester Centre for Genomic Medicine, St Mary's Hospital, Manchester University Hospitals NHS Foundation Trust, Oxford Road, Manchester, UK; Manchester Centre for Genomic Medicine, St Mary's Hospital, Manchester University Hospitals NHS Foundation Trust, Oxford Road, Manchester, UK; Manchester Centre for Genomic Medicine, St Mary's Hospital, Manchester University Hospitals NHS Foundation Trust, Oxford Road, Manchester, UK; Acute Medical Unit, Manchester Royal Infirmary, Manchester University Hospitals NHS Foundation Trust, Manchester, UK; Acute Medical Unit, Manchester Royal Infirmary, Manchester University Hospitals NHS Foundation Trust, Manchester, UK; Acute Medical Unit, Manchester Royal Infirmary, Manchester University Hospitals NHS Foundation Trust, Manchester, UK; Manchester Heart Centre, Manchester Royal Infirmary, Manchester University NHS Foundation Trust, Manchester, UK; Manchester Vascular Centre, Manchester Royal Infirmary, Manchester University NHS Foundation Trust, Manchester, UK; Acute Medical Unit, Manchester Royal Infirmary, Manchester University Hospitals NHS Foundation Trust, Manchester, UK; Biostatistics Collaboration Unit, Division of Population Health, Health Services Research & Primary Care, School of Health Sciences, The University of Manchester, Manchester, UK; Centre for Primary Care and Health Services Research, Division of Population Health, Health Services Research and Primary Care, School of Health Sciences, The University of Manchester, Manchester, UK; Manchester Centre for Health Economics, Division of Population Health, Health Services Research and Primary Care, School of Health Sciences, The University of Manchester, Manchester, UK; Manchester Centre for Health Economics, Division of Population Health, Health Services Research and Primary Care, School of Health Sciences, The University of Manchester, Manchester, UK; Manchester Centre for Genomic Medicine, St Mary's Hospital, Manchester University Hospitals NHS Foundation Trust, Oxford Road, Manchester, UK; Division of Evolution, Infection and Genomics, School of Biological Sciences, The University of Manchester, Manchester, UK

## Abstract

**Background:**

Pharmacogenetic-guided prescribing can be used to improve the safety and effectiveness of medicines. There are several approaches by which this intervention might be implemented in clinical practice, which will vary depending on the health system and clinical context.

**Aim:**

To understand the clinical utility of panel-based pharmacogenetic testing in patients admitted acutely to hospital and to establish variables that predict if an individual might benefit from the intervention.

**Design:**

A cross-sectional study recruiting patients admitted acutely to hospital.

**Methods:**

Participants underwent panel-based pharmacogenetic testing, and their genetic results were analysed in their context of the medicines they had been exposed to as an inpatient. The primary outcome was the proportion of patients with clinically actionable gene–drug interactions. Individual variables that predict the clinical utility of pharmacogenetic testing were established via logistic regression.

**Results:**

Genetic and prescribing data were available for 482 inpatients (55% male; median age 61.2 years; range: 18–96), 97.9% of whom carried a pharmacogenetic result of interest. During their admission, 79.5% of patients were exposed to a medicine for which there is pharmacogenetic prescribing guidance available. Just under one in seven individuals (13.7%) had a clinically actionable gene–drug interaction. Increasing age (>50 years) was positively correlated with the likelihood (2.7-fold increased risk) of having a clinically actionable interaction.

**Conclusions:**

These findings demonstrate the potential scale, and potential clinical utility, of pharmacogenetic testing as an intervention, highlighting the need to develop infrastructure to support healthcare professionals make use of this emerging tool.

## Introduction

There are many factors that influence an individual’s response to medicine, some of which can be used *a priori* to help guide prescribing. Variables such as weight, renal function, and allergy status are routinely considered during the prescribing process, with clinicians adjusting doses or selecting new medicines accordingly. One emerging variable increasingly considered is an individual’s genetic profile, a concept known as pharmacogenetics.^1^ Common genetic changes can impact response to frequently prescribed medicines, such as antidepressants, opioids, antiplatelets, statins and proton pump inhibitors (PPIs).^2–6^

In many countries, pharmacogenetic testing in specific clinical scenarios is now commonplace. Following guidance from the European Medicines Agency and a drug safety update published by the Medicines and Healthcare products Regulatory Agency, each year in the UK approximately 50 000 patients undergoing treatment for cancer with fluoropyrimidine chemotherapy agents are now tested for variants in *DPYD*.^7–9^ Individuals who carry two loss of function (LoF) variants in the *DPYD* gene (∼1 in 1000) are at risk of severe and potentially fatal toxicity if treated with these medicines, and therefore alternative agents should be selected. More recently, in July 2024, the National Institute for Health and Care Excellence has recommended that patients who have an ischaemic stroke or transient ischaemic attack, approximately 110 000 individuals a year in the UK, should be offered *CYP2C19* genotyping to guide antiplatelet therapy.^10^

Both these examples represent ‘reactive’ testing approaches, where variants in a single gene are genotyped in response to a clinical trigger to prescribe a medicine. Given the high population-frequency of clinically actionable pharmacogenetic variants, an increasing number of healthcare systems have begun to explore the use of panel-based genotyping.^11^^,^^12^ This involves testing individuals for many common pharmacogenetic variants at a set time, and then using that information to guide prescribing during future healthcare interactions. Although this approach has been explored in other health systems, mainly at single institutions, it has not yet been studied in the NHS, therefore relatively little is understood about the potential impact of this intervention on routine prescribing practice across a health system.^11–13^ There are ambitions within NHS strategy to integrate pharmacogenetic testing into routine practice, but how this might happen at scale is unclear, and the impacts of implementation on routine prescribing practice are uncertain.^14^^,^^15^ We aimed to investigate the potential clinical utility of implementing a panel-based pharmacogenetic test for patients admitted into an acute NHS hospital setting.

## Materials and methods

This study was designed to investigate how the introduction of panel-based pharmacogenetic-guided prescribing could impact routine prescribing practice in an acute NHS hospital setting, if existing pharmacogenetic guidance were adopted at scale.

### Study design

The Implementing Pharmacogenetics to Improve Prescribing (IPTIP) study was an investigator initiated, single-centre, cross-sectional study conducted in the NHS in England (ISRCTN 14050335). The study adhered to good clinical practice guidelines and the Declaration of Helsinki. All participants provided written informed consent prior to participation and the study received approval from the NHS research ethics committee (IRAS 305751) and the Human Research Authority.

### Participants and recruitment

Patients were eligible for recruitment if they were admitted as an inpatient to an acute medical or surgical ward at the Manchester University NHS Foundation Trust, 18 years of age or older, and had capacity to independently consent. To minimize sampling bias, patients were approached in a prespecified order, determined by a randomly generated list of beds, updated daily. Participants provided permission to access their medical records and provided either a blood or saliva sample for genotyping.

### Primary outcome

Whether patients had a clinically actionable gene–drug interaction.

### Secondary outcomes

Whether patients had clinically relevant gene–drug interactions, whether demographic variables were associated with the primary outcome, and the distribution of pharmacogenetic variation in the cohort.

### Genetic testing

Extracted DNA was genotyped using the Agena Veridose Core panel (Agena Bioscience, San Diego, USA) with Digital Droplet PCR (ddPCR) to confirm *CYP2D6* copy number (Supplementary Appendix S1). Genetic data (the genotype) were exported from the Agena platform and converted to metabolizer state (the phenotype), or functional status, for subsequent analysis using Stata V14.0 (StataCorp, College Station, TX, USA). This process was informed by guidance from the Clinical Pharmacogenetics Implementation Consortium (CPIC).^2^^,^^4–6^^,^^16–21^

### Data collection

Patient’s electronic health records were reviewed at the point of recruitment and after discharge. Each unique medicine that appeared on the patients’ electronic prescription chart during their inpatient stay was extracted, along with any recorded drug allergy, presenting complaint, and demographic data including age and sex. Ethnicity was self-reported.

### Establishing clinical utility

The potential impact of pharmacogenetic testing, had the data been available at the point of prescribing, was categorized as either ‘no impact’, ‘clinically relevant’ or ‘clinically actionable’ (Supplementary Appendix S2). A ‘clinically actionable’ interaction was defined as one where the recommended action within CPIC guidance was to alter the chosen medicine or dose. A ‘clinically relevant’ interaction was where the gene–drug interaction conferred increased risk of an adverse drug reaction (ADR) or reduced effectiveness, though the recommended action was to initiate therapy at a standard dose.

### Sample size

Participants were recruited over an 18-month window in an effort to reduce the impact of seasonal effects. The minimum sample size was chosen to identify the proportion of individuals with a clinically actionable or relevant variant with a prespecified desired precision of 0.05 and a 95% confidence interval. The formula $n=Z^{2}xPx(1-P)/e^{2}$ was used where *z* is a value from standard normal distribution corresponding to desired confidence level (*Z* = 1.96 for 95% confidence interval (CI)), *P* is expected true proportion, *e* is desired precision. An expected proportion of 40% was used, based on previous estimates from US-based studies.^13^ Using this calculation, the minimum required sample size was 369.

### Statistical analysis

Descriptive statistics were used to summarize demographic details, pharmacogenetic results, and clinical actionability. Fisher’s exact test was used to test for differences in the distribution of metabolizer states in different self-reported ethnic groups. Logistic regression analysis was used to examine the association between demographic and health-related variables with the presence of a clinically actionable variant as the dependent variable. Potential predictor variables were initially screened using univariate logistic regression, reporting odds ratios (ORs). Variables with a *P* values <0.20 in univariate analysis were included in a multivariate logistic regression model. A stepwise selection process was then used to select the final model. Model discrimination was assessed using the area under the receiver operating characteristic curve (AUC) and model calibration was examined with Pearson’s chi-squared and the Hosmer–Lemeshow goodness-of-fit tests. The likelihood ratio test was used to compare nested models from the stepwise procedure. ORs and 95% CIs were calculated to quantify the magnitude of associations. All statistical analysis was undertaken in Stata.

## Results

### Study population

The final study population included 511 participants recruited between 4 July 2022 and 1 January 2024 (Table 1). Of these, 56.2% of the population were male and the median age at recruitment was 62.1 (interquartile range 27.8 years). Approximately three-quarters of the population (74.6%) reported their ethnicity as White British, and 187 individuals (36.6%) had at least one recorded allergy. Prescribing data were available in the electronic healthcare records (EHRs) for 499 individuals, 97.6% of the population. Of those 499 patients, DNA could not be extracted from 18, leaving a final analysis cohort of 482 individuals with pharmacogenetic results (Supplementary Appendix S3).

**Table 1.** Participant characteristics

Characteristic	Study population	Analysis cohort
	(*n* = 511)	(*n* = 482)
Gender		
Male	287 (56.2%)	265 (55.0%)
Female	224 (43.8%)	217 (45.0%)
Age (years)		
Median age (IQR)	62.1 (45.5–73.3)	61.2 (44.2–72.3)
18–30	71 (13.9%)	70 (14.5%)
30–40	32 (6.3%)	30 (6.2%)
40–50	44 (8.6%)	44 (9.1%)
50–60	83 (16.2%)	81 (16.8%)
60–70	113 (22.1%)	104 (21.6%)
70–80	105 (20.5%)	99 (20.5%)
80–90	57 (11.2%)	48 (10.0%)
90+	6 (1.2%)	6 (1.2%)
Ethnicity		
White British/White Irish	381 (74.6%)	357 (74.1%)
Asian/Asian British	46 (9.0%)	42 (8.7%)
Black/African/Caribbean	38 (7.5%)	38 (7.9%)
Other white ethnic group	22 (4.3%)	22 (4.6%)
Other ethnic group	4 (0.8%)	4 (0.8%)
Mixed ethnicity	4 (0.8%)	4 (0.8%)
Not recorded	16 (3.1%)	15 (3.1%)
Recorded drug allergies^a^		
No (NKDA)	324 (63.4%)	305 (63.3%)
Yes	187 (36.7)	176 (36.5%)
1 allergy	133 (26.0%)	126 (26.1%)
2 allergies	43 (8.4%)	41 (8.5%)
3 allergies	10 (2.0%)	9 (1.9%)
4 allergies	1 (0.2%)	0 (0.0%)
Clinical presentation		
Medical	352 (68.9%)	345 (71.6%)
Cardiology	52 (10.2%)	50 (10.4%)
Dermatology	2 (0.4%)	2 (0.4%)
Endocrinology	24 (4.7%)	23 (4.8%)
Gastroenterology	57 (11.2%)	57 (11.8%)
Haematology	30 (5.9%)	30 (6.2%)
Infectious disease	24 (4.7%)	25 (5.2%)
Neurology	48 (9.4%)	48 (10.0%)
Psychiatry	1 (0.2%)	1 (0.2%)
Nephrology	11 (2.2%)	11 (2.3%)
Respiratory	83 (16.2%)	83 (17.2%)
Rheumatology	2 (0.4%)	2 (0.4%)
Toxicology	13 (2.5%)	13 (2.7%)
Surgical	104 (20.4%)	82 (17.0%)
Orthopaedic	10 (2.0%)	9 (1.9%)
Urology	6 (1.2%)	4 (0.8%)
Vascular	88 (17.2%)	69 (13.9%)
Not recorded	55 (10.8%)	55 (11.4%)

Percentages rounded to the nearest 0.1% and therefore may not sum to 100%.

a Drug allergies recorded in the patient’s electronic health record, indicating a contradiction to the prescription of a particular medicine.

IQR, interquartile range.

### Prescribing practice

**Figure 1.**
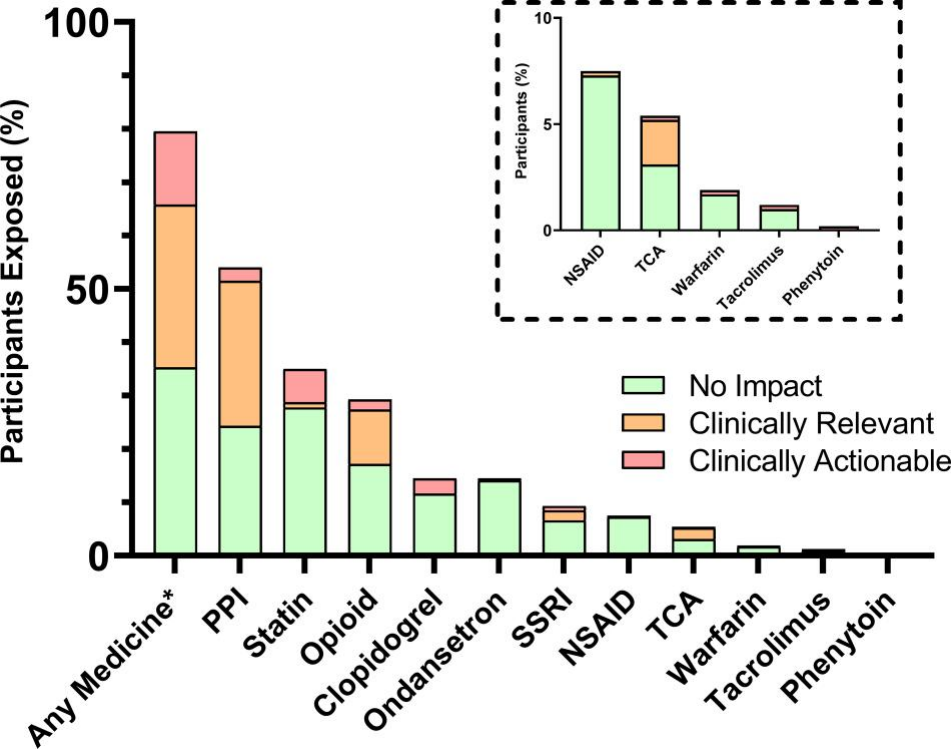
Prescribing practice and potential impact of pharmacogenetic results. The proportion of the population exposed to different groups of medicines with known gene–drug associations are displayed. The proportion of the population who had clinically relevant and clinically actionable gene–drug interactions are coloured in orange and red, respectively. Inset figure is an enlarged image showing the five least frequently prescribed medicines. NSAID, non-steroidal anti-inflammatory drug; PPI, proton pump inhibitor; SSRI, selective serotonin reuptake inhibitor. *Any medicine of the 11 medicine groups listed, for which pharmacogenetic prescribing guidance exists.

The mean total number of medicines a patient was prescribed across their admission was 13.5 (SD 8.21), with a range of 0–61. A majority of participants (79.5%) were exposed to a medicine during their admission for which CPIC guidance exists (Figure 1). Two hundred and fifty-three participants (52.5%) were exposed to two or more medicines for which CPIC guidance exists (Supplementary Appendix S4). The most frequently prescribed medicines were PPIs (52.5%), statins (34.9%), opioids (28.8%) and clopidogrel (14.5%) (Figure 1). Prescribing practice varied by sex, with men more frequently prescribed statins and women more likely to be prescribed TCAs (Supplementary Appendix S4). Rates of antidepressant prescribing were higher in women.

### Pharmacogenetic variation

**Figure 2.**
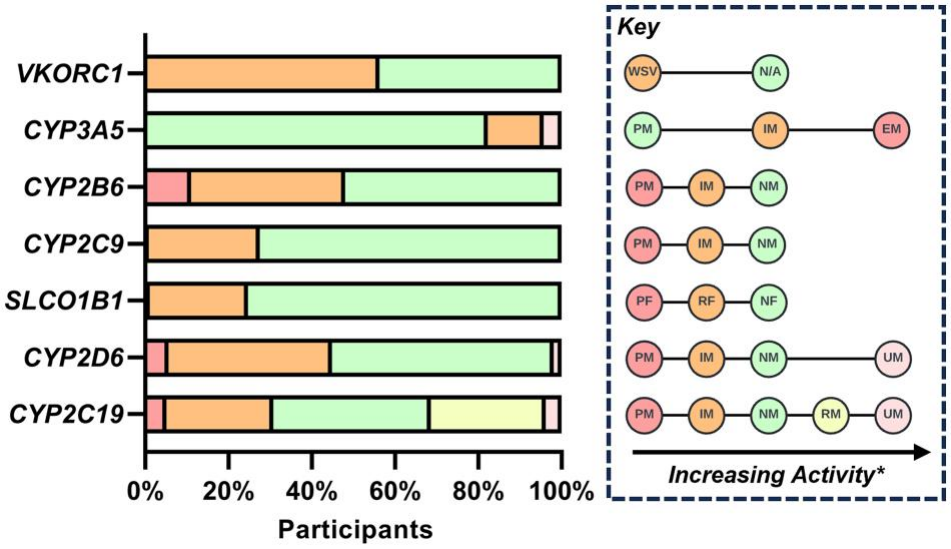
Pharmacogenetic variation across the study cohort. The frequency of each functional state across the study cohort within the seven target genes are shown in the stacked chart. Proportions represent the number of individuals with each functional state, divided by the total number of individuals who had a laboratory result for the relevant gene. The key on the right demonstrates the functional states across each gene, arranged by increasing activity from left to right. Further detail, including allele failure rate, is presented in Supplementary Appendix S3. *The *VKORC1* variant tested does not increase activity but increases sensitivity to warfarin. EM, extensive metabolizer; IM, intermediate metabolizer; NF, normal function; NM, normal metabolizer; PF, poor function; PM, poor metabolizer; RF, reduced function; RM, rapid metabolizer; UM, ultrarapid metabolizer; WSV, warfarin sensitivity variant (i.e. carrier of (c.-1639G>A)).

Almost all participants (97.9%) were found to carry at least one pharmacogenetic result of interest, conferring a ‘non-normal’ functional status in at least one of the seven targets assessed. The mean number of pharmacogenetic results of interest per patient was 2.7 (SD 1.14), with 122 individuals (25.3%) found to have four or more results of interest (Supplementary Appendix S4). There was considerable genetic variation across the cohort, with a large proportion of individuals carrying LoF alleles in key genes related to medicines metabolism (Figure 2). There were significant differences in the distribution of pharmacogenetic variation between ethnicities (Supplementary Appendix S5). *CYP2C19* LoF variants were more common in individuals of Asian ancestry (*P* = 0.001), while *CYP2D6* (*P* = 0.018), *VKORC1* (*P* = 0.005) and *SLCO1B1* (*P* = 0.029) variations were identified more frequently in individuals of White British ancestry.

### Clinical utility of pre-emptive pharmacogenetic panel testing

Across the cohort, 46.3% of participants had pharmacogenetic results related to a medicine they were exposed to during their admission (Figure 1). Just under one in seven individuals (13.7%) had a gene–drug interaction categorized as ‘clinically actionable’ meaning that had the results been available at the point of prescribing, an adjustment to dose or medicine selection would have been recommended as per CPIC guidance. Approximately one in three individuals (35.1%) had a gene–drug interaction considered ‘clinically relevant’, where there was a susceptibility to poor effectiveness or an adverse drug reaction, but an immediate prescribing adjustment was not indicated. Sixty-one individuals (12.7%) had two or more separate gene–drug interactions during their admission, and for 41 of these individuals (9.5%), a different gene was involved in one of the subsequent interactions.

The most frequent gene–drug interactions identified were related to opioids, clopidogrel, statins, and PPIs. Of the 70 individuals prescribed clopidogrel, 14 (20.0%) were *CYP2C19* LoF allele carriers meaning an alternative antiplatelet would have been recommended. 41% of all patients exposed to opioid analgesics during their admissions had a *CYP2D6* result, which might impact the effectiveness or safety of treatment. Within this group, 6.5% of patients were either ultrarapid or poor *CYP2D6* metabolizers, meaning guidance would be to avoid codeine and tramadol in this group entirely. Over one in five (21.1%) patients exposed to a statin carried at least one reduced function *SLCO1B1* allele, which could have been used to inform agent selection and dosing. Clinically actionable gene–drug interactions were identified in all medicine classes assessed, except for non-steroidal anti-inflammatory drugs.

### Predictors of clinical utility

Logistic regression modelling for the association between demographic variables and the primary outcome found that increasing age was a significant predictor of having a clinically actionable gene–drug interaction. The effect for age of having a clinically actionable drug–gene interaction conferred an OR (95% CI) = 1.02 (1.00–1.03) with a *P* values = 0.01 (Supplementary Appendix S6). The effect of patients aged over 50 years conferred an OR (95% CI) = 2.66 (1.32–5.38) with a *P* value = 0.006. Being male (OR 1.76, 95% CI 1.02–3.05) was the only other variable, which was significantly (*P* = 0.042) associated with a clinically actionable interaction (Supplementary Appendix S6). No other demographic variables were individually associated with a clinically actionable interaction.

A multivariate logistic regression model was developed, and the final iteration included age over 50 years, sex, ethnicity, total number of recorded allergies, and surgical admission (Supplementary Appendix S6). The multivariate model showed gender and age over 50 were associated with a clinically actionable interaction (gender: OR = 1.82, 95% CI = 1.03–3.22, *P* = 0.04; age over 50: OR = 2.15, 95% CI = 1.02–4.51, *P* = 0.04). Within this cohort, the model had satisfactory discrimination (AUC: 0.64) and calibration (Hosmer–Lemeshow test chi-squared = 6.34; Pearson’s chi-squared test = 38.8).

## Discussion

Genetics has traditionally been applied in healthcare in the diagnosis and management of rare disorders, defined as conditions that impact fewer than 1 in 2000 individuals.^22^ This study demonstrates the relative ubiquity of pharmacogenetics as an intervention, with the vast majority of the cohort (97.9%) found to carry at least one pharmacogenetic result of interest and 79.5% of the cohort exposed to a medicine for which pharmacogenetic prescribing guidance exists. The frequency of this variation, and the routine exposure to associated medicines, highlights the importance of panel-based pharmacogenetic testing and having the data available at the moment of prescribing.

The frequency at which pharmacogenetic data might be relevant in routine practice necessitates the development of a service that can facilitate the re-use of this information over time. One-quarter of patients were exposed to four or more medicines for which pharmacogenetic guidance is available. Asking clinicians to interpret traditional genetic test reports in the context of each of these prescribing decisions would be immensely challenging, creating increased workload and requiring a sustained educational programme. If pharmacogenetic data are to be truly integrated into routine practice, there has to be integration into EHRs, with any guidance contextualized as clinical decision support.^23^^,^^24^ This approach has been adopted by some healthcare organizations around the world, and surfacing these data within EHRs is entirely possible.^11^ However, ensuring pharmacogenetic results are available across an entire health system in both primary and secondary care, irrespective of geography or EHR vendor, is highly complex and will require significant investment.

As a secondary outcome, this study aimed to assess whether any demographic variables correlated with the likelihood of having a clinically actionable gene–drug interaction. In this cohort, logistic regression analysis found that patients over the age of 50 years had a 2.7-fold increase in the likelihood of a significant gene–drug interaction. This association is highly likely to be explained by the higher rates of prescribing in older age groups, increasing the likelihood of being exposed to a medicine for which pharmacogenetic prescribing guidance exists.^25^^,^^26^ Recognizing this pattern, some pharmacogenetic initiatives have developed eligibility criteria based on age, ranging from 50 to 65 years.^27–29^ In a resource-constrained environment, where testing is unlikely to be offered across an entire population, the findings in this project suggest age may be a useful factor in identifying who to prioritize for the intervention.

Other demographic variables, such as sex, ethnicity, and allergy status, were shown to marginally improve the overall discriminatory ability of a multivariate logistic model to identify those at increased risk of having a clinically actionable interaction. These findings do not support the triaging of access to pharmacogenetic testing based on sex or ethnicity. Rather, for the former, they serve to highlight differences in prescribing practice, with men more likely to be taking statins, PPIs and clopidogrel, with women more likely to be prescribed antidepressants. Regarding ethnicity, pharmacogenetic technologies, such as the one used in this study, are designed based on studies mainly undertaken in individuals of white European ancestry. As such, the lower frequency of relevant pharmacogenetic results in individuals not of White British background likely reflects the inadequacies of current testing panels in relation to ethnic diversity. This is a well-recognized phenomenon in genomics generally, and any implementation strategy must endeavour to adopt and develop ethnically inclusive testing and recruitment strategies.^30–32^ Without this, the testing, guidance, and associated algorithms will remain biased, widening health inequalities.

A limitation of this work is that in practice, like any individual biomarker, pharmacogenetic results would not typically be used in isolation to make a prescribing decision. Rather, results are routinely contextualized, taking into consideration the broader clinical picture, polypharmacy, and whether the patient is already established on treatment. As such, there is no certainty that clinically actionable interactions would have resulted in a prescribing adjustment. Finally, this study makes no distinction between pre-existing and new prescriptions. Although pharmacogenetic-guided medicines reconciliation processes have been trialled successfully in the past, this represents a distinct implementation challenge from the use of this data to guide new prescriptions.^33^^,^^34^

## Conclusion

Pharmacogenetic variation is present at high frequencies across the population and exposure to medicines with pharmacogenetic guidelines is common. If this data was available pre-emptively, pharmacogenetic-guided prescribing could be relevant to a large proportion of the population at multiple times across their admission. Implementing pharmacogenetics in practice will require the development of informatic solutions to make sense of the results, to avoid healthcare professionals being over burdened by the scale and complexity of information.

## Supplementary Material

hcae200_Supplementary_Data

## Data Availability

The data that support the findings of this study are openly available in the ISRCTN registry at https://doi.org/10.1186/ISRCTN14050335, reference number ISRCTN1405033.
