# Supplementary material for: Pre-emptive pharmacogenetic testing in the acute hospital setting: a cross-sectional study
Source: QJM. 2024 Oct 17;118(3):154–60. doi: 10.1093/qjmed/hcae200 (PMC12051386; doi:10.1093/qjmed/hcae200)
Supplement: hcae200_Supplementary_Data [file hcae200_supplementary_data.docx]

**Pre-Emptive Pharmacogenetics in The Acute Hospital Setting: A Cross Sectional Study**

**Contents**

1. **Appendix 1: Pharmacogenetic Variants Tested**
2. **Appendix 2: Clinically Actionable & Clinically Relevant Definitions**
3. **Appendix 3: Pharmacogenetic test results by gene across the cohort (n=482) & associated failure rate**
4. **Appendix 4: Supplementary Tables**
5. **Appendix 5: Summary of pharmacogenetic test results & breakdown by ethnicity**
6. **Appendix 6: Logistic Regression Analysis**

**Appendix 1: Pharmacogenetic Variants Tested**

Table S1. Pharmacogenetic Variants Tested

| **Gene** | **Nucleotide Change** | **Amino Acid Change / Star Allele** | **dbSNP RS#** |
| --- | --- | --- | --- |
| *ABCB1* | c.3435C>T | p.I1145= | rs1045642 |
| *APOE* | c.388T>C | p.C130R | rs429358 |
|  | c.526C>T | p.R176C | rs7412 |
| *COMT* | c.472G>A | p.V158M | rs4680 |
| *CYP1A2* | g.-3860G>A | *1C | rs2069514 |
|  | g.-163C>A | *1F | rs762551 |
|  | g.-729C>T | *1K | rs12720461 |
|  | g.3533G>A | *7 | rs56107638 |
|  | g.558C>A | *11 | rs72547513 |
| *CYP2B6* | c.516G>A | *6 | rs3745274 |
|  | c.983T>C | *18 | rs28399499 |
| *CYP2C19* | c.681G>A | *2 | rs4244285 |
|  | c.636G>A | *3 | rs4986893 |
|  | c.1A>G | *4 | rs28399504 |
|  | c.1297C>T | *5 | rs56337013 |
|  | c.395G>A | *6 | rs72552267 |
|  | g.19294T>A | *7 | rs72558186 |
|  | c.358T>C | *8 | rs41291556 |
|  | g.-806C>T | *17 | rs12248560 |
| *CYP2C9* | c.430C>T | *2 | rs1799853 |
|  | c.1075A>C | *3 | rs1057910 |
|  | c. 1076T>C | *4 | rs56165452 |
|  | c.1080C>G | *5 | rs28371686 |
|  | c.818delA | *6 | rs9332131 |
|  | c.449G>A | *8 | rs7900194 |
|  | c.1003C>T | *11 | rs28371685 |
|  | c.1465C>T | *12 | rs9332239 |
|  | c.269T>C | *13 | rs72558187 |
|  | c.485C>A | *15 | rs72558190 |
| *CYP2D6* | g.2850C>T | *2, multiple | rs16947 |
|  | g.4180G>C | *2, multiple | rs1135840 |
|  | g.2549delA | *3 | rs35742686 |
|  | g.1846G>A | *4 | rs3892097 |
|  | g.1707delT | *6 | rs5030655 |
|  | g.2935A>C | *7 | rs5030867 |
|  | g.1758G>T | *8 | rs5030865 |
|  | g.2613_2615delAGA (g.2615_2617delAAG) | *9 | rs5030656 |
|  | g.100C>T | *10, multiple | rs1065852 |
|  | c.883G>C | *11 | rs201377835 |
|  |  |  | rs5030863 |
|  | g.124G>A | *12 | rs5030862 |
|  | g.1758G>A | *14 | rs5030865 |
|  | g.137-138insT | *15 | rs72549357 |
|  | g.1023C>T | *17 | rs28371706 |
|  | c.dup4125-4133 | *18 | dup4125_4133 |
|  | c.2539_2542delAACT | *19 | rs72549353 |
|  | c.1973_1974insG | *20 | rs72549354 |
|  | g 3183G>A | *29 | rs59421388 |
|  | g.2988G>A | *41 | rs28371725 |
|  |  | CNV | CNV |
| *CYP3A4* | c.664T>C | *2 | rs55785340 |
|  | c.566 T>C | *17 | rs4987161 |
|  | g.15389C>T | *22 | rs35599367 |
| *CYP3A5* | g.27289C>A | *2 | rs28365083 |
|  | g.6986A>G | *3 | rs776746 |
|  | c.624G>A | *6 | rs10264272 |
|  | g.27131_27132insT | *7 | rs41303343 |
| *DRD2* | c.2137G>A | p.E713K / Taq1A | rs1800497 |
| *F2* | c.*97G>A | p.G20210A | rs1799963 |
| *F5* | c.1601G>A | p.R506Q | rs6025 |
| *GLP1R* | c.780A>C/T | p.L260F | rs1042044 |
|  | c.502G>A/C | p.G168R/G168S | rs6923761 |
|  | c.510-1135T>G |  | rs2300615 |
| *MTHFR* | c.665C>T | p.A222V | rs1801133 |
|  | c.1286A>C | p.E429A | rs1801131 |
| *OPRM1* | c.118A>G | p.A118G | rs1799971 |
| *PNPLA5* | c.608-169G>A |  | rs5764010 |
| *SLCO1B1* | c.521T>C | p.V174A | rs4149056 |
| *SULT4A1* | c.743-374A>G |  | rs763120 |
| *VKORC1* | c.-1639G>A | 1639 G>A | rs9923231 |

**Appendix 2 - Clinically Actionable & Clinically Relevant Definitions & Classification**

Recommendation tables were extracted from CPIC guidance (appendix 2) and each recommendation was coded by the authors as either “no impact”, “clinically relevant”, or “clinically actionable”. These three groups were defined as:

- **No Impact (Green)** – Recommendations which advise standard dosing.
- **Clinically Relevant (Orange)** – Recommendations which advise starting with a standard dose but advise there is an increased risk of poor response or side effects, and that treatment adjustments may be required.
- **Clinically Actionable (Red)** – Recommendations which advise dose adjustment or selecting an alternative medicine.

**Appendix 2.1 Clopidogrel [CYP2C19]**

| **CYP2C19 Phenotype** | **Therapeutic Recommendation** | **Study Classification** |
| --- | --- | --- |
| Ultrarapid | If considering clopidogrel, use at standard dose (75 mg/day) | No Impact |
| Rapid | If considering clopidogrel, use at standard dose (75 mg/day) | No Impact |
| Normal | If considering clopidogrel, use at standard dose (75 mg/day) | No Impact |
| Intermediate | Avoid clopidogrel if possible. Use prasugrel or ticagrelor at standard dose if no contraindication | Clinically Actionable |
| Poor | Avoid clopidogrel if possible. Use prasugrel or ticagrelor at standard dose if no contraindication | Clinically Actionable |

**Appendix 2.2 Opioids (Tramadol & Codeine) [CYP2D6]**

| **CYP2D6 Phenotype** | **Therapeutic Recommendation** | **Study Classification** |
| --- | --- | --- |
| Ultrarapid | Avoid codeine use because of potential for serious toxicity. If opioid use is warranted, consider a non-tramadol opioid. | Clinically Actionable |
| Normal | Use codeine label recommended age-specific or weight-specific dosing. | No Impact |
| Intermediate | Use codeine label recommended age-specific or weight-specific dosing. If no response and opioid use is warranted, consider a non-tramadol opioid. | Clinically Relevant |
| Poor | Avoid codeine use because of possibility of diminished analgesia. If opioid use is warranted, consider a non-tramadol opioid. | Clinically Actionable |

**Appendix 2.3 Tacrolimus [CYP3A5]**

| **CYP3A5 Phenotype** | **Therapeutic Recommendation** | **Study Classification** |
| --- | --- | --- |
| Extensive  (Expressor) | Increase starting dose 1.5–2 times recommended starting dose.d Total starting dose should not exceed 0.3 mg/kg/day. Use therapeutic drug monitoring to guide dose adjustments. | Clinically Actionable |
| Intermediate (Expressor) | Increase starting dose 1.5–2 times recommended starting dose.a Total starting dose should not exceed 0.3 mg/kg/day. Use therapeutic drug monitoring to guide dose adjustments. | Clinically Actionable |
| Poor (Non-Expressor) | Initiate therapy with standard recommended dose. Use therapeutic drug monitoring to guide dose adjustments. | No impact |

**Appendix 2.4 Warfarin [VKORC1 *and* CYP2C9]**

| **Genotype** | **Therapeutic Recommendation** | **Study Classification** |
| --- | --- | --- |
| *VKORC1* -1639G>A and CYP2C9 Loss of Function Allele Carrier | Calculate dose based on validated published pharmacogenetic algorithms | Clinically Actionable |
| Any other combination of *VKORC1* and genotypes | Dose clinically | No Impact |

**Appendix 2.5 Phenytoin [CYP2C9]**

| **CYP2C9 Phenotype** | **Therapeutic Recommendation** | **Study Classification** |
| --- | --- | --- |
| Normal Metaboliser | No adjustments needed from typical dosing strategies. Subsequent doses should be adjusted according to therapeutic drug monitoring, response and side effects | No Impact |
| *CYP2C9* Intermediate Metaboliser (Activity Score 1.5) | No adjustments needed from typical dosing strategies. Subsequent doses should be adjusted according to therapeutic drug monitoring, response and side effects | No Impact |
| *CYP2C9* Intermediate Metaboliser (Activity Score 1.0) | For first dose, use typical initial or loading dose. For subsequent doses, use ~ 25% less than typical maintenance dose. Subsequent doses should be adjusted according to therapeutic drug monitoring, response and side effects. | Clinically Actionable |
| *CYP2C9* Poor Metaboliser | For first dose, use typical initial or loading dose. For subsequent doses use ~ 50% less than typical maintenance dose. Subsequent doses should be adjusted according to therapeutic drug monitoring, response, and side effects. | Clinically Actionable |

Footnote = HLA-B*15:02 not tested. Guidance assumed patient is HLA-B*15:02 negative.

**Appendix 2.6 Selective Serotonin Reuptake Inhibitors (SSRIs) [*CYP2C9*, *CYP2D6 & CYP2B6*]**

**Appendix 2.6.1 Citalopram & CYP2C19**

| **CYP2C19 Phenotype** | **Therapeutic Recommendation** | **Study Classification** |
| --- | --- | --- |
| Ultrarapid | Consider a clinically appropriate alternative antidepressant not predominantly metabolized by CYP2C19. If citalopram or escitalopram are clinically appropriate, and adequate efficacy is not achieved at standard maintenance dosing, consider titrating to a higher maintenance dose | Clinically Actionable |
| Rapid | Initiate therapy with recommended starting dose. If patient does not adequately respond to recommended maintenance dosing, consider titrating to a higher maintenance dose or switching to a clinically appropriate alternative antidepressant not predominantly metabolized by CYP2C19 | Clinically Relevant |
| Normal | Initiate therapy with recommended starting dose | No Impact |
| Intermediate | Initiate therapy with recommended starting dose. Consider a slower titration schedule and lower maintenance dose than normal metabolizers | Clinically Relevant |
| Poor | Consider a clinically appropriate antidepressant not predominantly metabolized by CYP2C19. If citalopram or escitalopram are clinically appropriate, consider a lower starting dose, slower titration schedule, and 50% reduction of the standard maintenance dose as compared with normal metabolizers | Clinically Actionable |

**Appendix 2.6.2 Paroxetine & CYP2D6**

| **CYP2D6 Phenotype** | **Therapeutic Recommendation** | **Study Classification** |
| --- | --- | --- |
| Ultrarapid | Select alternative drug not predominantly metabolized by CYP2D6 | Clinically Actionable |
| Normal | Initiate therapy with recommended starting dose | No Impact |
| Intermediate | Consider a lower starting dose and slower titration schedule as compared with normal metabolizers | Clinically Relevant |
| Poor | Consider a 50% reduction in recommended starting dose, slower titration schedule, and a 50% lower maintenance dose as compared with normal metabolizers | Clinically Actionable |

**Appendix 2.6.3.1 Sertraline & CYP2C19**

| **CYP2C19 Phenotype** | **Therapeutic Recommendation** | **Study Classification** |
| --- | --- | --- |
| Ultrarapid | Initiate therapy with recommended starting dose | No Impact |
| Rapid | Initiate therapy with recommended starting dose | No Impact |
| Normal | Initiate therapy with recommended starting dose | No Impact |
| Intermediate | Initiate therapy with recommended starting dose. Consider a slower titration schedule and lower maintenance dose than CYP2C19 normal metabolizers | Clinically Relevant |
| Poor | Consider a lower starting dose, slower titration schedule, and 50% reduction of standard maintenance dose as compared with CYP2C19 normal metabolizers or select a clinically appropriate alternative antidepressant not predominantly metabolized by CYP2C19 | Clinically Actionable |

**Appendix 2.6.3.2 Sertraline & CYP2B6**

| **CYP2B6 Phenotype** | **Therapeutic Recommendation** | **Study Classification** |
| --- | --- | --- |
| Ultrarapid | Initiate therapy with recommended starting dose | No Impact |
| Rapid | Initiate therapy with recommended starting dose | No Impact |
| Normal | Initiate therapy with recommended starting dose | No Impact |
| Intermediate | Initiate therapy with recommended starting dose. Consider a slower titration schedule and lower maintenance dose than CYP2B6 normal metabolizers | Clinically Relevant |
| Poor | Consider a lower starting dose, slower titration schedule, and 25% reduction of standard maintenance dose as compared with CYP2B6 normal metabolizers or select a clinically appropriate alternative antidepressant not predominantly metabolized by CYP2B6 | Clinically Actionable |

**Appendix 2.6.4 Venlafaxine**

| **CYP2D6 Phenotype** | **Therapeutic Recommendation** | **Study Classification** |
| --- | --- | --- |
| Ultrarapid | No action recommended based on genotype for venlafaxine because of minimal evidence regarding the impact on efficacy or side effects | No Impact |
| Normal | Initiate therapy with recommended starting dose | No Impact |
| Intermediate | No action recommended based on genotype for venlafaxine because of minimal evidence regarding the impact on efficacy or side effects | No Impact |
| Poor | Consider a clinically appropriate alternative antidepressant not predominantly metabolized by CYP2D6 | Clinically Actionable |

**Appendix 2.7 Tricyclic Antidepressants**

**Appendix 2.7.1 Tricyclic Antidepressants (Amitriptyline, Nortriptyline & Clomipramine) & *CYP2D6***

| **CYP2D6 Phenotype** | **Therapeutic Recommendation** | **Study Classification** |
| --- | --- | --- |
| Ultrarapid | Avoid tricyclic use due to potential lack of efficacy. Consider alternative drug not metabolized by CYP2D6. If a TCA is warranted, consider titrating to a higher target dose (compared to normal metabolizers). Utilize therapeutic drug monitoring to guide dose adjustments. | Clinically Actionable |
| Normal | Initiate therapy with recommended starting dose. | No Impact |
| Intermediate | Consider a 25% reduction of recommended starting dose. Utilize therapeutic drug monitoring to guide dose adjustments. | Clinically Relevant |
| Poor | Avoid tricyclic use due to potential for side effects. Consider alternative drug not metabolized by CYP2D6. If a TCA is warranted, consider a 50% reduction of recommended starting dose. Utilize therapeutic drug monitoring to guide dose adjustments. | Clinically Actionable |

**Footnote:** Guidance applied to all TCAs.

**Appendix 2.7.2 Tricyclic Antidepressants (Amitriptyline & Clomipramine) & *CYP2C19***

| **CYP2C19 Phenotype** | **Therapeutic Recommendation** | **Study Classification** |
| --- | --- | --- |
| Ultrarapid | Avoid tertiary amine use due to potential for sub-optimal response. Consider alternative drug not metabolized by CYP2C19. TCAs without major CYP2C19 metabolism include the secondary amines nortriptyline and desipramine. If a tertiary amine is warranted, utilize therapeutic drug monitoring to guide dose adjustments. | Clinically Actionable |
| Rapid | Avoid tertiary amine use due to potential for sub-optimal response. Consider alternative drug not metabolized by CYP2C19. TCAs without major CYP2C19 metabolism include the secondary amines nortriptyline and desipramine. If a tertiary amine is warranted, utilize therapeutic drug monitoring to guide dose adjustments. | Clinically Actionable |
| Normal | Initiate therapy with recommended starting dose. | No Impact |
| Intermediate | Initiate therapy with recommended starting dose. | No Impact |
| Poor | Avoid tertiary amine use due to potential for sub-optimal response. Consider alternative drug not metabolized by CYP2C19. TCAs without major CYP2C19 metabolism include the secondary amines nortriptyline and desipramine. For tertiary amines, consider a 50% reduction of the recommended starting dose. Utilize therapeutic drug monitoring to guide dose adjustments | Clinically Actionable |

**Footnote:** Guidance applies to the tertiary amines amitriptyline and clomipramine only.

**Appendix 2.8 Statins [*SLCO1B1* & *CYP2C9*]**

**Appendix 2.8.1 Simvastatin & *SLCO1B1***

| **SLCO1B1 Phenotype** | **Therapeutic Recommendation** | **Study Classification** |
| --- | --- | --- |
| Normal Function | Prescribe desired starting dose and adjust doses based on disease-specific guidelines | No Impact |
| Decreased Function | Prescribe an alternative statin depending on the desired potency | Clinically Actionable |
| Poor Function | Prescribe an alternative statin depending on the desired potency | Clinically Actionable |

**Appendix 2.8.2 Atorvastatin & *SLCO1B1***

| **SLCO1B1 Phenotype** | **Therapeutic Recommendation** | **Study Classification** |
| --- | --- | --- |
| Normal Function | Prescribe desired starting dose and adjust doses based on disease-specific guidelines | No Impact |
| Decreased Function | Prescribe ≤40 mg as a starting dose and adjust doses of atorvastatin based on disease-specific guidelines. Prescriber should be aware of possible increased risk for myopathy especially for 40-mg dose. If dose >40 mg needed for desired efficacy, consider combination therapy (i.e., atorvastatin plus nonstatin guideline-directed medical therapy) | Clinically Relevant |
| Poor Function | Prescribe ≤20 mg as a starting dose and adjust doses of atorvastatin based on disease-specific guidelines. If dose >20 mg is needed for desired efficacy, consider rosuvastatin or combination therapy (i.e., atorvastatin plus nonstatin guideline- directed medical therapy)3 | Clinically Actionable |

**Appendix 2.8.3 Fluvastatin & *SLCO1B1***

| **SLCO1B1 Phenotype** | **Therapeutic Recommendation** | **Study Classification** |
| --- | --- | --- |
| Normal Function | Prescribe desired starting dose and adjust doses based on disease-specific guidelines | No Impact |
| Decreased Function | Prescribe desired starting dose and adjust doses of fluvastatin based on disease-specific guidelines. Prescriber should be aware of possible increased risk for myopathy especially for doses >40 mg per day | Clinically Relevant |
| Poor Function | Prescribe ≤40 mg per day as a starting dose and adjust doses of fluvastatin based on disease- specific guidelines. If patient is tolerating 40 mg per day but higher potency is needed, a higher dose (>40 mg) or an alternative statin (see Figure 1 for recommendations for alternative statins) or combination therapy (i.e., fluvastatin plus nonstatin guideline-directed medical therapy) could be considered. Prescriber should be aware of possible increased risk for myopathy with fluvastatin especially with doses >40 mg per day | Clinically Actionable |

**Appendix 2.8.4 Fluvastatin & *CYP2C9***

| **CYP2C9 Phenotype** | **Therapeutic Recommendation** | **Study Classification** |
| --- | --- | --- |
| Normal Metaboliser | Prescribe desired starting dose and adjust doses of fluvastatin based on disease-specific guidelines | No Impact |
| Intermediate Metaboliser | Prescribe ≤40 mg per day as a starting dose and adjust doses of fluvastatin based on disease-specific guidelines. If dose >40 mg needed for desired efficacy, consider an alternative statin or combination therapy (i.e., fluvastatin plus nonstatin guideline-directed medical therapy) | Clinically Relevant |
| Poor Metaboliser | Prescribe ≤20 mg per day as a starting dose and adjust doses of fluvastatin based on disease-specific guidelines. If dose >20 mg needed for desired efficacy, consider an alternative statin or combination therapy (i.e., fluvastatin plus nonstatin guideline- directed medical therapy) | Clinically Actionable |

**Appendix 2.8.5 Rosuvastatin & *SLCO1B1***

| **SLCO1B1 Phenotype** | **Therapeutic Recommendation** | **Study Classification** |
| --- | --- | --- |
| Normal Function | Prescribe desired starting dose and adjust doses based on disease-specific guidelines | No Impact |
| Decreased Function | Prescribe desired starting dose and adjust doses of rosuvastatin based on disease-specific and population-specific guidelines | No Impact |
| Poor Function | Prescribe ≤20 mg as a starting dose and adjust doses of rosuvastatin based on disease-specific and population-specific guidelines If dose >20 mg needed for desired efficacy, consider combination therapy (i.e., rosuvastatin plus nonstatin guideline- directed medical therapy) | Clinically Actionable |

**Appendix 2.9 Non-Steroidal Anti-Inflammatory Drugs (NSAIDs) (Ibuprofen, piroxicam & celecoxib) [CYP2C9]**

**Appendix 2.9.1 NSAIDs (Ibuprofen & Celecoxib) & *CYP2C9***

| **CYP2C9 Phenotype** | **Therapeutic Recommendation** | **Study Classification** |
| --- | --- | --- |
| Normal Metaboliser | Initiate therapy with recommended starting dose. | Not Actionable |
| Intermediate Metaboliser – Activity Score 1.5 | Initiate therapy with recommended starting dose. | Not Actionable |
| Intermediate Metaboliser – Activity Score 1.0 | Initiate therapy with lowest recommended starting dose. Titrate dose upward to clinical effect or maximum recommended dose with caution due to increased risk of toxicity. | Clinically Relevant |
| Poor Metaboliser | Initiate therapy with 25–50% of the lowest recommended starting dose. Titrate dose upward to clinical effect or 25–50% of the maximum recommended dose with caution. In accordance with the prescribing information, use the lowest effective dosage for shortest duration consistent with individual patient treatment goals. Upward dose titration should not occur until after steady-state is reached | Clinically Actionable |

**Appendix 2.9.2 Piroxicam & *CYP2C9***

| **CYP2C9 Phenotype** | **Therapeutic Recommendation** | **Study Classification** |
| --- | --- | --- |
| Normal Metaboliser | Initiate therapy with recommended starting dose. | Not Actionable |
| Intermediate Metaboliser – Activity Score 1.5 | Initiate therapy with recommended starting dose. | Not Actionable |
| Intermediate Metaboliser – Activity Score 1.0 | Choose an alternative therapy not metabolized by CYP2C9 or not significantly impacted by CYP2C9 genetic variants in vivo or choose an NSAID metabolized by CYP2C9 but with a shorter half-life | Clinically Relevant |
| Poor Metaboliser | Choose an alternative therapy not metabolized by CYP2C9 or not significantly impacted by CYP2C9 genetic variants in vivo or choose an NSAID metabolized by CYP2C9 but with a shorter half-lifeconsistent with individual patient treatment goals. Upward dose titration should not occur until after steady-state is reached | Clinically Actionable |

**Appendix 2.10 Proton Pump Inhibitors (Omeprazole, lansoprazole, pantoprazole) [*CYP2C19*]**

| **CYP2C19 Phenotype** | **Therapeutic Recommendation** | **Study Classification** |
| --- | --- | --- |
| Ultrarapid | Increase starting daily dose by 100%. Daily dose may be given in divided doses. Monitor for efficacy | No Impact |
| Rapid | Initiate standard starting daily dose. Consider increasing dose by 50–100% for the treatment of Helicobacter pylori infection and erosive esophagitis. Daily dose may be given in divided doses. Monitor for efficacy. | Clinically Relevant |
| Normal | Initiate standard starting daily dose. Consider increasing dose by 50–100% for the treatment of H. pylori infection and erosive esophagitis. Daily dose may be given in divided doses. Monitor for efficacy | No Impact |
| Intermediate | Initiate standard starting daily dose. For chronic therapy (> 12 weeks) and efficacy achieved, consider 50% reduction in daily dose and monitor for continued efficacy | Clinically Relevant |
| Poor | Initiate standard starting daily dose. For chronic therapy (> 12 weeks) and efficacy achieved, consider 50% reduction in daily dose and monitor for continued efficacy | Clinically Relevant |

**Appendix 2.11 Ondansetron [*CYP2D6*]**

| **CYP2D6 Phenotype** | **Therapeutic Recommendation** | **Study Classification** |
| --- | --- | --- |
| Ultrarapid | Select alternative drug not predominantly metabolized by CYP2D6 (i.e., granisetron) | Clinically Actionable |
| Normal | Initiate therapy with recommended starting dose. | No Impact |
| Intermediate | Insufficient evidence demonstrating clinical impact based on CYP2D6 genotype. Initiate therapy with recommended starting dose | No Impact |
| Poor | Insufficient evidence demonstrating clinical impact based on CYP2D6 genotype. Initiate therapy with recommended starting dose | No Impact |

**Appendix 3: Pharmacogenetic test results by gene across the cohort (n=482) & associated failure rate**


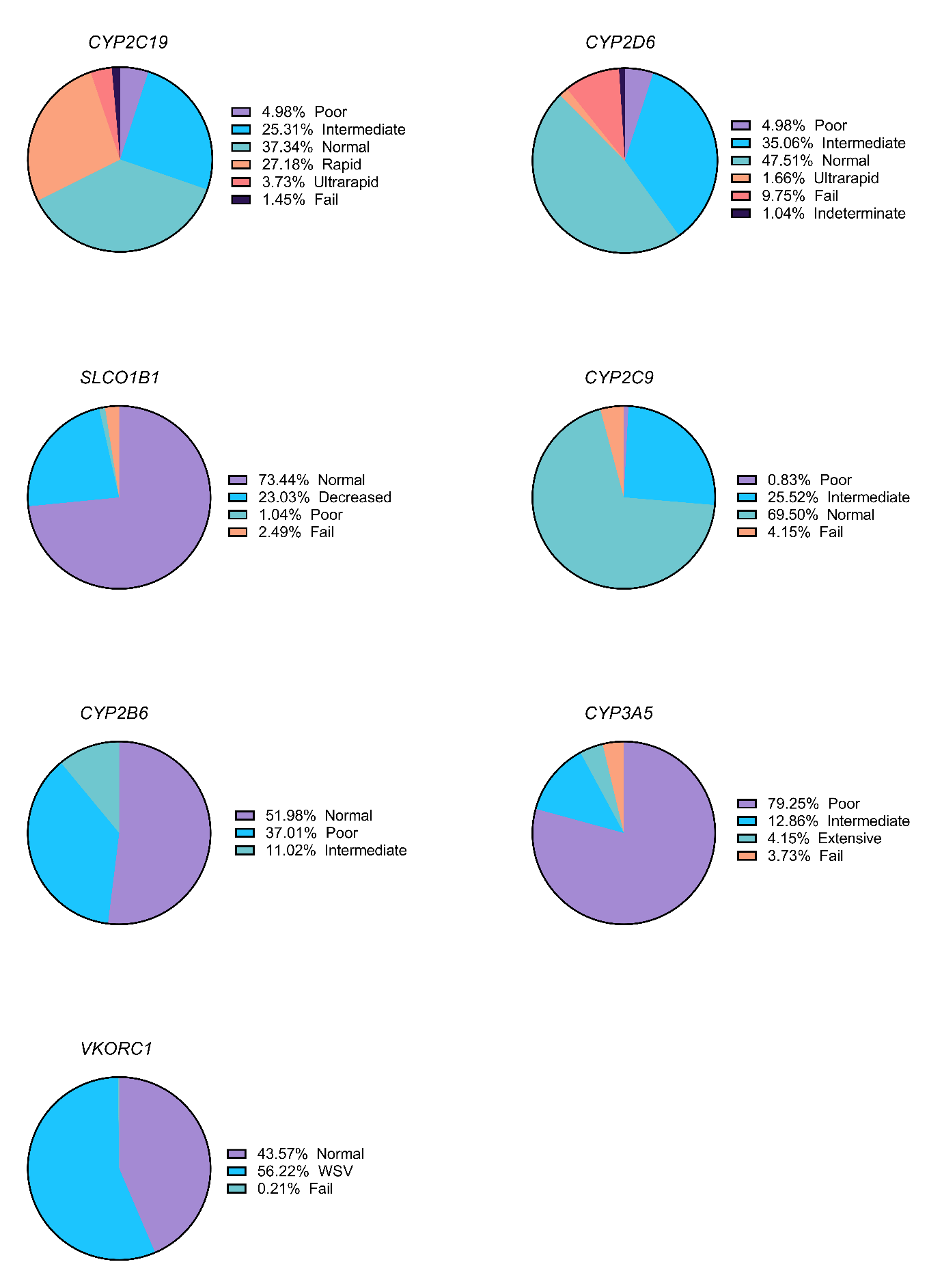


**Figure S1. Spread of Pharmacogenetic Variation Across Cohort**

**Appendix 4: Supplementary Tables**

**Table S2.**

| Number of Medicines with Pharmacogenetic Guidance Available | 0 | 1 | 2 | 3 | 4 | 5 | 6 |
| --- | --- | --- | --- | --- | --- | --- | --- |
| Patients | 99 (20.5%) | 130  (27.0%) | 112  (23.2%) | 97  (20.1%) | 34  (7.1%) | 8  (1.7%) | 2  (0.4%) |

**Footnote:** Table showing the number (%) of individuals who were exposed to different numbers of medicines for which pharmacogenetic guidance is available. For example, 99 (20.5%) individuals were exposed to no medicines for which pharmacogenetic guidance existed, whereas 97 (20.1%) were exposed to three medicines with pharmacogenetic guidance.

**Table S3**

| Number of Clinically Relevant/Actionable Interactions | 0 | 1 | 2 | 3 | 4 |
| --- | --- | --- | --- | --- | --- |
| Patients | 269 (55.8%) | 152  (31.5%) | 47  (9.8%) | 10  (2.1%) | 4  (0.8%) |

**Footnote:** Table showing the number (%) of individuals who had different numbers of clinically actionable or relevant gene-drug interactions. For example, 269 (55.8%) individuals had no clinically relevant or actionable gene-drug interactions, whereas 10 (2.1%) individuals had 3 clinically relevant or actionable gene-drug interactions.

**Table S4. Prescribing by Sex**

|  | **Female** | **Male** | p value |
| --- | --- | --- | --- |
| **Opioids** | 55 | 84 | 0.131 |
|  | 25.35% | 31.70% |  |
| **TCA** | 18 | 8 | **0.014** |
|  | 8.29% | 3.02% |  |
| **Warfarin** | 3 | 6 | 0.523 |
|  | 1.38% | 2.26% |  |
| **Statin** | 63 | 103 | **0.027** |
|  | 29.03% | 38.87% |  |
| **SSRIs** | 22 | 22 | 0.527 |
|  | 10.14% | 8.30% |  |
| **PPI** | 106 | 145 | 0.201 |
|  | 48.85% | 54.72% |  |
| **Clopidogrel** | 27 | 43 | 0.299 |
|  | 12.44% | 16.23% |  |
| **Tacrolimus** | 2 | 4 | 0.695 |
|  | 0.92% | 1.51% |  |
| **Ondansetron** | 31 | 39 | 0.999 |
|  | 14.29% | 14.72% |  |
| **Phenytoin** | 0 | 1 | 0.999 |
|  | 0.00% | 0.38% |  |
| **NSAID** | 17 | 18 | 0.725 |
|  | 0 | 1 |  |

**Footnote =** p value calculated using Fishers Exact Test.

**Appendix 5: Summary of pharmacogenetic test results & breakdown by ethnicity**

In the Cytochrome P450 genes, LoF alleles were identified most frequently in *CYP2B6* (48.0% of participants), *CYP2D6* (44.9%), and *CYP2C19* (30.8%). Two LoF alleles, conferring a poor metaboliser phenotype and indicating severely reduced enzyme activity, were found at rates of 11.0%, 5.6% and 5.1% in *CYP2B6*, *CYP2D6* and *CYP2C19,* respectively. Copy number variants in *CYP2D6* were identified in 8.9% of the study population, 3.1% of which were deletions and 5.8% were duplications. Decreased function variants in *SLCO1B1*, increasing the likelihood of statin induced myopathy, were found in just under one quarter (24.7%) of the cohort, and 1.1% carried two of these alleles, conferring a poor *SLCO1B1* functional status. The common *VKORC1* LoF allele was found in 56.3% of individuals, and 28.4% of participants were either poor or intermediate *CYP2C9* metabolisers. 17.7% of individuals had a CYP3A5 expressor genotype.

**Table S4. CYP2C19 Status Across Ethnic Groups**

*Fishers Exact Test

**Table S5. CYP2D6 Status Across Ethnic Groups**

**Table S6. CYP2C9 Status Across Ethnic Groups**

**Table S7. SLCO1B1 Function Across Ethnic Groups**

**Table S8. CYP3A5 Status Across Ethnic Groups**

**Table S9. VKORC1 Result Across Ethnic Groups**

**Table S10. CYP2B6 Function Across Ethnic Groups**

**Appendix 6: Logistic Regression Analysis**

As per the methodology, pre-defined predictor variables were initially screened using univariate logistic regression, reporting odds ratio. The dependent variable of interest was the presence of a clinically actionable gene-drug interaction, treated as a binary outcome. The variables of interest included in the logistic regression analysis, and their rationale for inclusion, are presented below.

| Variable | Variable Type | Rationale for Inclusion |
| --- | --- | --- |
| Age | Continuous | Previous studies have shown that increasing age is associated with increased exposure to medicines |
| Age ≥ 50 | Binary | Previous implementation programmes have used age 50 as part of eligibility criteria for pharmacogenetic programmes. The NHS’ “Well Person” checks are also available from this age (with some geographical variation). |
| Sex | Binary | To assess whether prescribing is impacted by sex |
| Any Recoded Allergy | Binary | Allergy status may correlate with clinically actionable gene-drug interactions, providing a basis for some of the previous reactions. |
| Total Number of Allergies | Continuous | Allergy status may correlate with clinically actionable gene-drug interactions, providing a basis for some of the previous reactions. |
| White British Ethnicity | Binary | Pharmacogenetic variation is distributed differently across different ethnicities (and most work has been undertaken in white populations), which may impact chance of detecting a clinically actionable interaction |
| Asian Ethnicity | Binary | Pharmacogenetic variation is distributed differently across different ethnicities (and most work has been undertaken in white populations), which may impact chance of detecting a clinically actionable interaction |
| Black Ethnicity | Binary | Pharmacogenetic variation is distributed differently across different ethnicities (and most work has been undertaken in white populations), which may impact chance of detecting a clinically actionable interaction |
| Surgical Admission | Binary | Exposure to different medicines may vary based on presenting complaint and management |
| Medical Admission | Binary | Exposure to different medicines may vary based on presenting complaint and management |

Outcomes from univariate analysis of each of the above variables is shown below (Age as a continuous variable is also presented graphically):

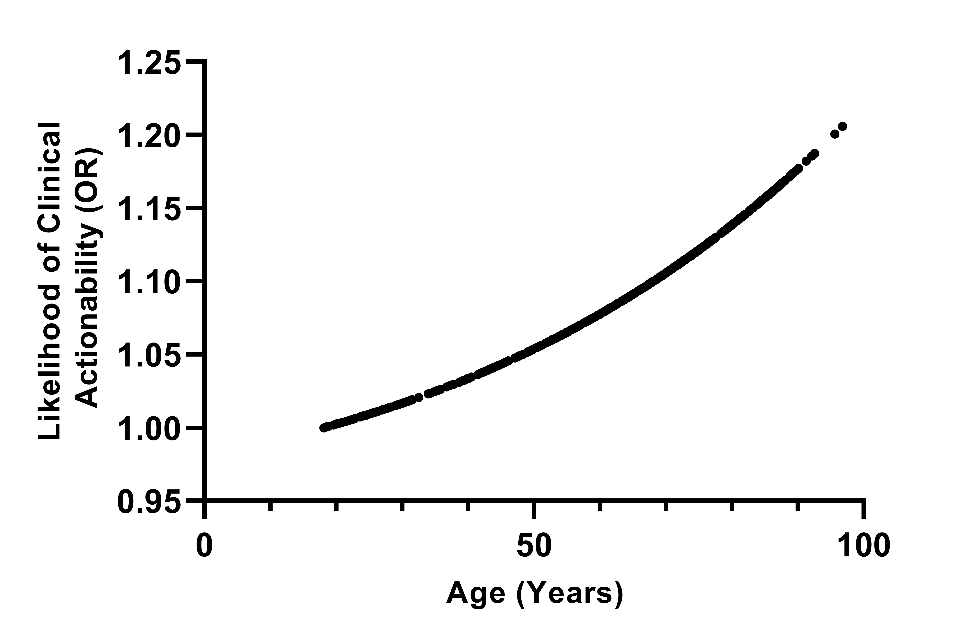


**Figure S2. Increase in likelihood of a clinically actionable interaction with age.**

Increasing age (as a continuous variable and when made binary (age>50) and male gender were associated with having a statistically significantly increased chance of having a clinically actionable gene-drug interaction.

Variables with a p-value<0.20 in univariate analysis were included in a multivariate logistic regression model. A stepwise selection process (using backwards selection) was then used to select the final model.

These include:

- Age over 50
- Sex
- Number of allergies
- White British Ethnicity
- Surgical Admission

Clearly related variables (i.e. age and age over 50) were not included in the same multivariate logistic regression analysis. Model discrimination was assessed using the area under the receiver operating characteristic curve (AUC) and model calibration was examined with Pearson’s chi-squared and the Hosmer-Lemeshow goodness-of-fit tests. The likelihood ratio test was used to compare nested models from the stepwise procedure. The final model is shown below.

**Final Model**

| N = 482 | Odds ratio | p-value | 95% CI |
| --- | --- | --- | --- |
|  |  |  |  |
| Male | 1.82 | 0.040 | 1.03 to 3.22 |
| Age > 50 | 2.15 | 0.043 | 1.02 to 4.51 |
| Non-white British ethnicity | 0.71 | 0.343 | 0.35 to 1.45 |
| Number of allergies | 1.29 | 0.154 | 0.91 to 1.83 |
| Surgical admission | 1.20 | 0.582 | 0.62 to 2.32 |
|  |  |  |  |

**Figure S3.** Final model receiver operating characteristic curve

The final model had an AUC of 0.638. Pearson’s, Hosmer-Lemeshow and likelihood ratio tests all agreed that the full model (which included all potential covariates) provided the best fit.

| **Model** | **Covariates** | **Pearson's^1^** | **Hosmer-Lemeshow^1^** | **Likelihood Ratio^1^** | **AUC** |
| --- | --- | --- | --- | --- | --- |
| 1 | Gender, age over 50, WBE, no. of allergies, surgical admission | 38.8 (0.302) | 6.34 (0.610) | - | 0.638 |
| 2 | Gender, age over 50, WBE, no. of allergies | 19.7 (0.542) | 6.60 (0.471) | 0.30 (0.585) | 0.639 |
| 3 | Gender, age over 50, no. of allergies | 7.8 (0.734) | 4.71 (0.452) | 1.30 (0.552) | 0.637 |
| 4 | Gender, age over 50 | 0.8 (0.371) | 0.80 (0.670) | 3.44 (0.629) | 0.629 |

**Footnote:** Summary of outputs from the stepwise procedure. ^1^Figures are given as value of chi-square test statistic (p-value). WBE = White British ethnicity
